# Ultrasound Control of Pickering Emulsion-Based Capsule Preparation

**DOI:** 10.3390/s24175710

**Published:** 2024-09-02

**Authors:** Filip Ratajczak, Bassam Jameel, Rafał Bielas, Arkadiusz Józefczak

**Affiliations:** Faculty of Physics, Adam Mickiewicz University in Poznań, Uniwersytetu Poznańskiego 2, 61-614 Poznań, Poland; filip.ratajczak@amu.edu.pl (F.R.); bassam.jameel@amu.edu.pl (B.J.); arkadiusz.jozefczak@amu.edu.pl (A.J.)

**Keywords:** ultrasound, magnetic nanoparticles, Pickering emulsion, capsules

## Abstract

Capsules with microparticle shells became of great interest due to their potential in many fields. Those capsules can be fabricated at high temperatures from particle-stabilized emulsions (Pickering emulsions) by sintering together particles that cover droplets. One of the problems with such an approach is accurately controlling whether particles are already sintered and creating the rigid capsule shell of a capsule. Here, we propose using a non-destructive ultrasound method for monitoring Pickering emulsion-based capsules prepared using heating under an alternating magnetic field. The polyethylene microparticles that were responsive to temperatures higher than 112 °C were used as droplet stabilizers together with iron oxide nanoparticles. During the coalescence of the droplets, facilitated by an external electric field, the ultrasonic attenuation increased, giving evidence that the ultrasound method detects structural changes in Pickering emulsions. The main change was the difference in the droplets’ size, which was also observed via optical microscopy. The attenuation of ultrasound increased even more when measured after magnetic heating for the same concentration of particle stabilizers. Simultaneously, the values of ultrasonic velocity did not exhibit similar variety. The results show that the values of the attenuation coefficient can be used for a quantitative evaluation of the capsule formation process.

## 1. Introduction

Colloidal capsules are small capsules, in the order of micrometers, whose coatings are made of microparticles or nanoparticles. Their structure provides the separation of the substance enclosed inside them from the external environment. This allows for the storage of active substances that would undergo undesirable processes such as denaturation or the influence of the pH of the external environment [1].

The composition of capsule coating is closely related to the method of their production and subsequent application. They can be made of various materials, including polymers like polyethylene or polystyrene [2] and more biocompatible substances, such as gold, capable of interacting with living organisms without causing harmful effects [3]. Among the types of colloidal capsules described in the literature, there are also those made of iron–platinum (FePt) nanoparticles [4], silicon-based polymer nanocomposites [5,6], and pristine silicon nanoparticles [7]. Moreover, capsules have been prepared based on metal–organic frameworks [8], using carbon nanotubes [9,10], and polymer microprisms [11], and even bionanoparticles based on biological materials such as viruses [12,13] or proteins [14]. More complex stabilizers, such as polymer bubbles filled with gold nanoparticles, were also presented as promising coating materials [15].

Regardless of the preparation method, the material of the coating must provide durability and resistance to the undesirable effects of external factors. Such a rigid and durable capsule coating can be broken in a controlled manner using an external stimulus such as ultraviolet [16,17], electric or magnetic fields [18], high temperature [19], or a high-intensity ultrasonic wave [20,21]. This results in releasing the enclosed substance and its use according to the planned purpose. Colloidal capsules, therefore, have great potential for use in medicine, food, or the pharmaceutical industry. However, their use is still limited, among other reasons, because of difficulties in scaling up their production.

**Figure 1:**
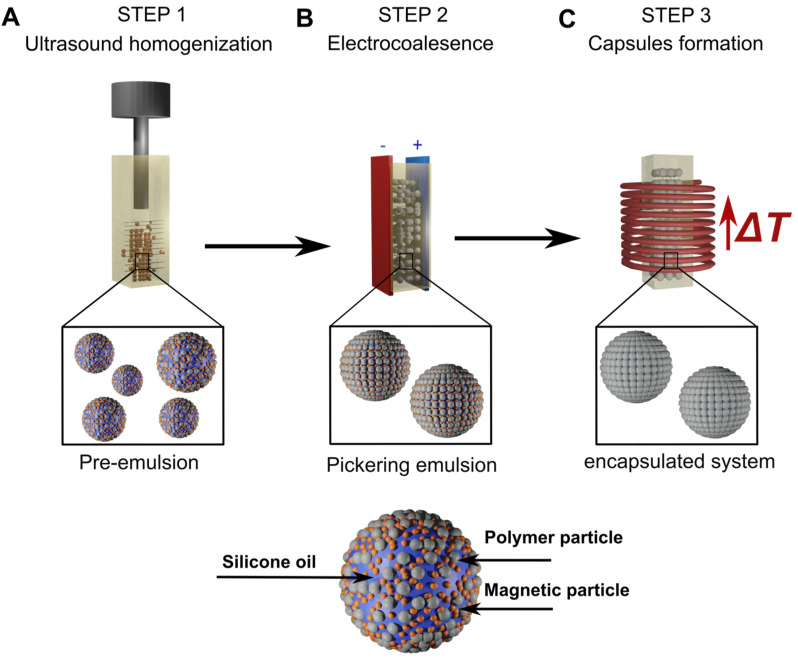
Scheme of the step–by–step process of capsule formation. (**A**) Ultrasonic homogenization is used to form emulsion, and (**B**) the DC electric field is applied to facilitate the formation of fully covered droplets. (**C**) The temperature increase of the emulsion under influence of an alternating magnetic field results in the fabrication of capsulated droplets. Blue spheres represent droplets of the dispersed phase (silicone oil), while smaller, gray, and red spheres represent polymer and magnetic particles, respectively. As a result of sintering, the polymer particles fuse, and the space between them (size of pores) decreases.

There are various methods of producing colloidal capsules, including: layer-by-layer technique for preparing thin films [22], method based on the use of amphiphilic particles with respect to both phases of colloidal systems with an attached polymer [23], method based on loading liposome shells with nanoparticles [24], and, finally, production of colloidal capsules from emulsion droplets stabilized by solid particles, i.e., Pickering droplets [2]. Colloidal capsules can be obtained by sintering the stabilizing particles present on the surface of Pickering droplets at high temperatures. These particles, under the influence of high temperatures, combine, creating a rigid coating on the surface of the droplets, thus transforming them into colloidal capsules upon cooling. As a result, a rigid structure is obtained around the droplets, protecting the substance contained inside the droplets from external factors. The preparation of emulsion and transformation of Pickering droplets into colloidal capsules is schematically presented in Figure 1.

Various methods of heating the system can be used to obtain the appropriate temperature necessary to sinter the particles, for example, more conventional microwave or magnetic heating. The properties of colloidal capsules can be modified by incorporating materials such as magnetic nanoparticles into their coating. The resulting structures are subject to the influence of an external magnetic field, allowing for the control and manipulation of capsule position and heating. 

Regardless of the strategy used to fabricate colloidal capsules, it is difficult to assess whether they have formed a rigid shell around the droplet. When the sintering of the individual particles in the shell is applied, the information on whether the particles have already sintered is crucial. Besides the direct observation of the structure that could be beneficial in some situations for large structures, one of the previously proposed methods was the optical measurements of droplets and capsules exposed to an external, constant electric field [2]. Because of the opacity of concentrated emulsion, especially when black and brown particles such as magnetite are in use, the dilution of the system might be necessary. Additionally, the deformation in response to electric stress highly depends on a droplet dimension. It cannot be observed for droplets of the size of tens of micrometers and smaller. To test the capsulation of smaller droplets, another method is required. Here, we propose utilizing the non-destructive ultrasound method.

Ultrasound testing is widely used in scientific and industrial applications such as the determination of the size of the objects dispersed in the liquid [25], control over the destabilization of colloidal systems, for instance, the sedimentation speed of the particles when suspended in the liquid phase [26], and evaluation of the elastic properties of particle suspension [27]. The main features of the ultrasound approach are robustness and the lack of required sample preparation. One should remember, however, that the description of ultrasound propagation in complex multi-phase systems, such as Pickering droplets (and resulting capsules), is mathematically challenging and, foremost, demands the values of the properties of the phases [28]. 

In this work, the effectiveness of measurements of acoustic parameters, specifically propagation velocity and attenuation of the ultrasonic waves, was checked to control the production process of colloidal capsules. Capsules were fabricated from Pickering emulsions stabilized by combining polyethylene and magnetic particles. In the process, magnetic heating was used to provide sufficient temperature elevation of the system.

## 2. Results 

### 2.1. Frequency Dependence of Ultrasound Velocity for Emulsions and Capsules

**Figure 2:**
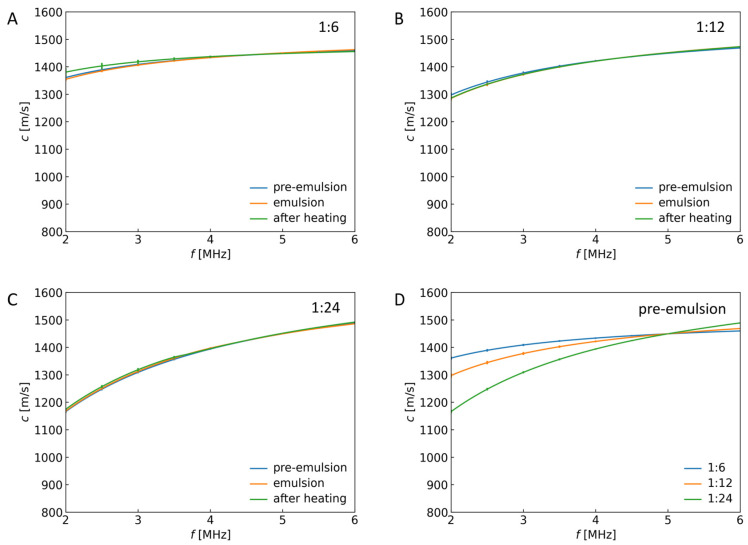
Ultrasound propagation velocity in the function of frequency in different systems for (**A**) 1:6, (**B**) 1:12, and (**C**) 1:24 polymer particle mass to silicone oil mass ratios. (**D**) Ultrasound propagation velocity versus frequency for different ratios of polymer particle mass to silicone oil mass in pre-emulsion.

**Figure 3:**
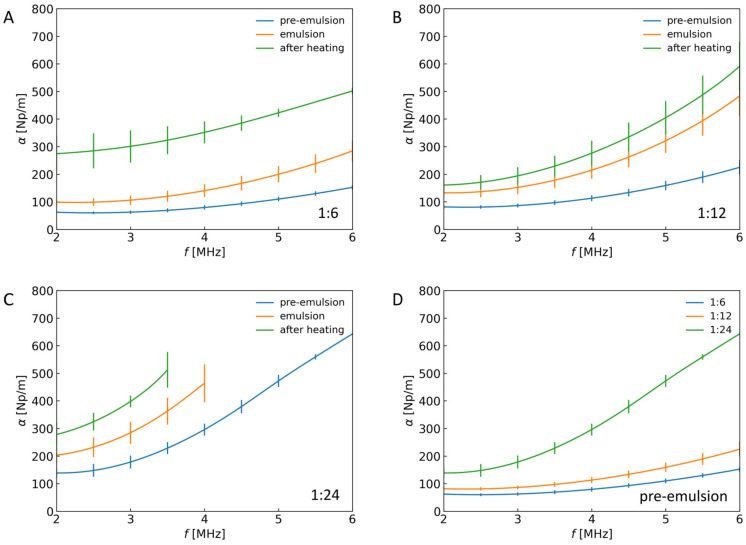
Ultrasound attenuation coefficient in the function of frequency in different systems for (**A**) 1:6, (**B**) 1:12, and (**C**) 1:24 polymer particle mass to silicone oil mass ratios. (**D**) Ultrasound attenuation coefficient versus frequency for different ratios of particle mass to silicone oil mass in pre-emulsion.

**Figure 5:**
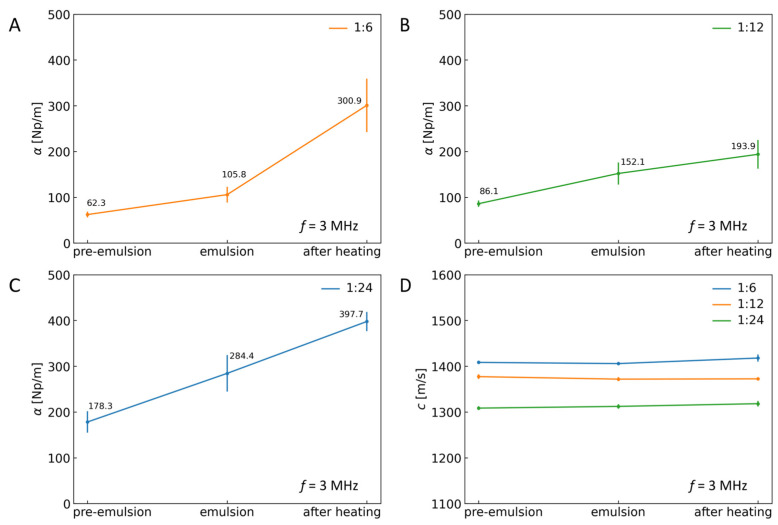
Ultrasound attenuation coefficient in different systems and for particle mass to silicone oil mass ratio: (**A**) 1:6, (**B**) 1:12, and (**C**) 1:24. (**D**) Ultrasound propagation velocity in different systems and for different ratios. Parameters were measured at frequency *f* = 3 MHz.

After each capsule formation stage, the ultrasound velocity and attenuation were measured using the methods described in Section 4.3. Three samples were prepared for each polymer particle mass to silicone oil mass ratio, and ultrasonic measurements were conducted. The results were later averaged from 3 measurements, collected in charts, and shown in Figure 2 and Figure 3. Figure 2 presents ultrasound propagation velocity as a function of frequency for different mass ratios of polymer particles to silicone oil. The ratios of polymer particle mass to silicone oil mass affected the ultrasound velocity value, especially for lower frequencies (Figure 2D). However, subjecting the pre-emulsion to a constant electric field to transform it into a stable emulsion and then heating it in an alternating magnetic field for creating capsules did not result in a significant change in ultrasound velocity (Figure 2A–C). This shows that the ultrasound velocity is insensitive to structural changes induced by capsule formation and cannot be used to control Pickering emulsion-based capsule preparation, even though it is well-known that the ultrasound velocity depends on the bulk compressibility of the system. The velocity values depend on the adiabatic compressibility, $\beta_{s}$, from the well-known Newton–Laplace formula $c=\frac{1}{\sqrt{\beta_{s}\rho }}$, where *ρ* is the density. Apparently, during the fabrication process of capsules (first under DC electric field, then AC magnetic field), these parameters change simultaneously, giving rise to no observable difference in propagation velocity when the concentration of stabilizing material remains constant. The same result will also be shown for the single frequency of the ultrasound wave (Figure 5D).

### 2.2. Frequency Dependence of Ultrasound Attenuation for Emulsions and Capsules

The situation is different in the case of ultrasound attenuation. During the analysis of the measurement results, an increase in the determined values of attenuation coefficients could be observed for each subsequent stage of the production of capsules from the Pickering emulsion, as shown in Figure 3.

**Figure 4:**
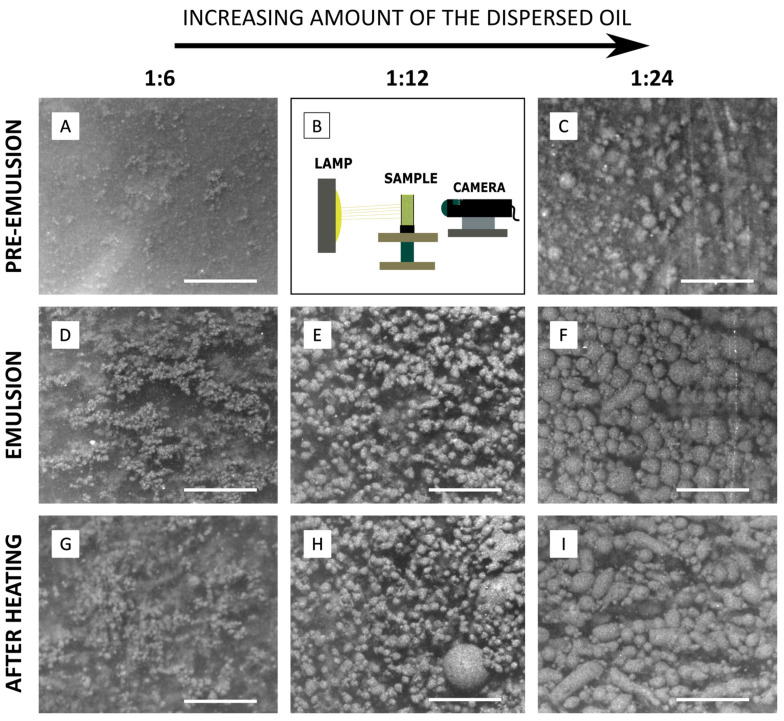
Optical microscopic images of analyzed systems for different ratios of polymer particle mass to silicone oil mass for: pre-emulsion—(**A**) 1:6, (**C**) 1:24 ratio; emulsion—(**D**) 1:6, (**E**) 1:12, (**F**) 1:24 ratio; emulsion after heating—(**G**) 1:6, (**H**) 1:12, (**I**) 1:24 ratio. Additionally, panel (**B**) provides a schematic drawing of the optical setup. The scale bar is 500 µm.

The DC electric field was applied during the first stage (conversion of the pre-emulsion into an emulsion). The electro-coalescence process occurred for each sample for the same time (15 min). The mean droplet size increased during the electro-coalescence, and the number of the droplets decreased. The small, densely dispersed droplets coalesced into several large drops with increased particle coverage, forming a stable Pickering emulsion. As previously shown [29], this should be accompanied by an increase in the attenuation of the ultrasonic wave, which is consistent with the results in Figure 3 and Figure 4. The role of droplet size is also illustrated in Figure 3D, which shows the ultrasound attenuation coefficient versus frequency for different ratios of polymer particle mass to silicone oil mass in pre-emulsion. As the amount of silicone oil in the system increases, the droplet size increases because the amount of stabilizing particles is the same, and this causes an increase in absorption. The value of the ultrasonic attenuation coefficient was the largest for the sample with a 1:24 ratio because large emulsion droplets formed, contributing to higher ultrasound wave attenuation.

In the next stage, which involved heating the emulsion in an alternating magnetic field, polymer particles partially melted on the droplet surface. After cooling the system, a rigid shell of sintered particles was formed. As shown in Figure 3, the formation of the rigid shell process is also accompanied by an increase in the ultrasound attenuation coefficient, observed for all ratios of polymer particle mass to silicone oil mass. 

From our previous works, when the polyethylene and polystyrene microparticles were used solely to stabilize emulsion droplets under a DC electric field, we assume here that the particle shell around the droplets before capsule formation consists of a monolayer of particles [30]. On the other hand, we showed before [28] that magnetic nanoparticles cover the droplets forming the aggregated structures. Based on the contrast in volume fractions between magnetic and polymer particles used in the current study and the size difference, where the polymer particles diameter (10–90 μm) and the magnetic diameter 157.5 nm ± 3.7 nm, we could anticipate the presence of the particle shell around the capsules of the size of particles. 

It is worth noting that in Figure 3, we observed the apparent influence of the distortion of calculated absorption spectra due to the low amplitude of the analyzed ultrasound pulses. It resulted in a narrower frequency range of analyzed results. It was also why we did not present the attenuation amplitude spectrum in the wider range of frequencies for the most attenuating systems (with the highest content of the droplets, 1:24). The existing artifact in the curve stemmed from the problem with an accurate analysis of pulses of low amplitudes. 

### 2.3. Optical Microscopy Images of Emulsions and Capsules Prepared via Magnetic Heating

Due to the limitation of direct observation of the process of capsule preparation from magnetite–polyethylene Pickering emulsions as templates, we showed above the possibility of using ultrasound waves to characterize the fabricated capsules. However, mainly when the content of the droplets in the system is lower, optical microscopy imaging could reveal some features of the tested systems, e.g., Pickering droplet deformations induced by an external electric field [2]. In this work, microscopic observations obtained and presented in Figure 4 are congruent with the results of ultrasonic measurements, revealing changes in the size and shape of the droplets. The presented images show the appearance of tested systems at each stage of measurements for different ratios of polymer particle mass to silicone oil mass. The droplets electro-coalesced and grew to sizes a few times larger than the mean droplet size of the pre-emulsion, which was also indicated by the difference in ultrasound velocity and attenuation in investigated systems (Section 2.1 and Section 2.2). 

### 2.4. Ultrasound Velocity and Attenuation Coefficients for a Single Frequency

The experimental values of ultrasound velocity and attenuation coefficient in the function of frequency could provide crucial information on the system when analyzed with the corresponding scattering theories, such as the Anson–Chivers core–shell model used recently to analyze the size of Pickering droplets prepared similarly to the current paper [28]. In practice, the single value should be sufficient to characterize the system, particularly for fast in-line testing. That is why ultrasound propagation velocity and attenuation coefficient differences for different systems and ratios of particle mass to silicone oil mass were compared at the single frequency of 3 MHz. The frequency was chosen based on Fast Fourier transform modules of the ultrasonic pulses recorded in analyzed systems. 

As one can see, the attenuation coefficient derived in this way also follows the trends described above, i.e., the magnetic heating influence, the value of ultrasound attenuation regardless of the content of particles in the tested sample, and varying droplet sizes (Figure 5A–C). However, due to the complexity of the studied system (a three-phase system after exposition to the high temperature increase), the data obtained for the single frequencies should be compared with the attenuation spectra presented in Figure 3. It should be done using the proposed approach based on ultrasound sensors. 

## 3. Discussion 

Results of previous investigations on the use of ultrasonic waves for monitoring changes during magnetic heating in analogical systems of oil-in-oil Pickering emulsions stabilized solely by magnetite nanoparticles [31] show that there was no significant change in values of ultrasound propagation velocity and attenuation coefficient before and after the use of heating on the system. Obtained changes in the values of those parameters in our work may suggest that properties of objects forming an emulsion (e.g., Pickering droplets) shifted due to changes induced by heating. We assume that temperature elevation is sufficient to surpass the transition temperature of polyethylene microparticles and allow them to sinter together on the surface of droplets. After cooling, they create rigid shells of colloidal capsules with magnetic nanoparticles blended in the structure. Fabricating capsules from emulsion droplets as templates exhibits another important feature: the oil core could incorporate species (such as drugs) when mixed simply during the emulsification process. Provided that the species are not fragile to higher temperatures (that can be locally increased much in the vicinity of magnetic nanoparticles), they are protected by the surrounding shell. 

Figure 2 and Figure 5D present the results that allow us to observe a noticeable difference in ultrasound propagation velocity between different ratios of particle mass to silicone oil mass. Similar differences were not distinguished between different systems for the same ratios, where values of ultrasound propagation velocity were comparable in the analyzed frequency range. Thus, more than ultrasound propagation velocity measurement is needed to be a sufficient indicator of the encapsulation process. However, because of the lack of a clear difference in the values of the velocity of the ultrasound propagating through the complex multi-phase system at different stages of preparation (from ultrasonic homogenization to prepare pre-emulsion to capsule formation under an alternating magnetic field, see Figure 5D), the information on ultrasound velocity could be used to monitor the temperature during magnetic heating as the temperature correlates well with the ultrasound velocity [32]. It would be more accurate than the IR camera monitoring that, although easy, can detect temperature changes only at the surface of the liquid medium. 

The ultrasound attenuation coefficient in the analyzed frequency range changes depending on the type of system and particle mass to silicone oil mass ratios, as shown in Figure 3 and Figure 5B. The highest values of ultrasound attenuation coefficient were observed in emulsions after heating for all particle mass to silicone oil mass ratios. As presented in Figure 5A, C, the ultrasound attenuation is higher for the emulsion and emulsion after heating when compared to the pre-emulsion. The emulsions have higher particle coverage and a bigger droplet radius due to electro-coalescence. In other words, the ultrasound technique was successfully used to detect the change in material state. The results showed an increase in the ultrasound attenuation with the change in the n-hexadecane from the crystalized point to the melting point [33]. Moreover, the low-intensity ultrasound wave was used as an in-line sensor for detecting the production process of the polymer material. Polymer melting and crystallization have contributed to the changed ultrasound attenuation value due to different loss scattering after the observed transitions [34]. Despite no existing data on control of the transition process of polymer particles stabilizing Pickering droplets, we obtained higher attenuation after sintering the particle shell. That could suggest that a similar structural transition occurred also in this case. The simultaneous use of data on ultrasonic velocity and the change in attenuation of ultrasound when capsules are formed could be used to determine encapsulation. This way, we could obtain two separate quantitative parameters informing about the capsule formation process via magnetic heating from the same recorded ultrasonic pulses.

Optical images indicate that some droplets undergo deformation during the heating process, creating bigger and non-spherical capsules. The difference is particularly visible for 1:12 polymer particle mass to silicone oil mass ratios (Figure 4E,H) and 1:24 (Figure 4F,I). The presented optical images also clearly show that the Pickering emulsions stabilized with magnetic and non-magnetic particles could be prepared using the proposed method and are not destroyed when exposed to temperature elevation due to magnetic heating. It is aligned with our previous results [31]. 

**Figure 6:**
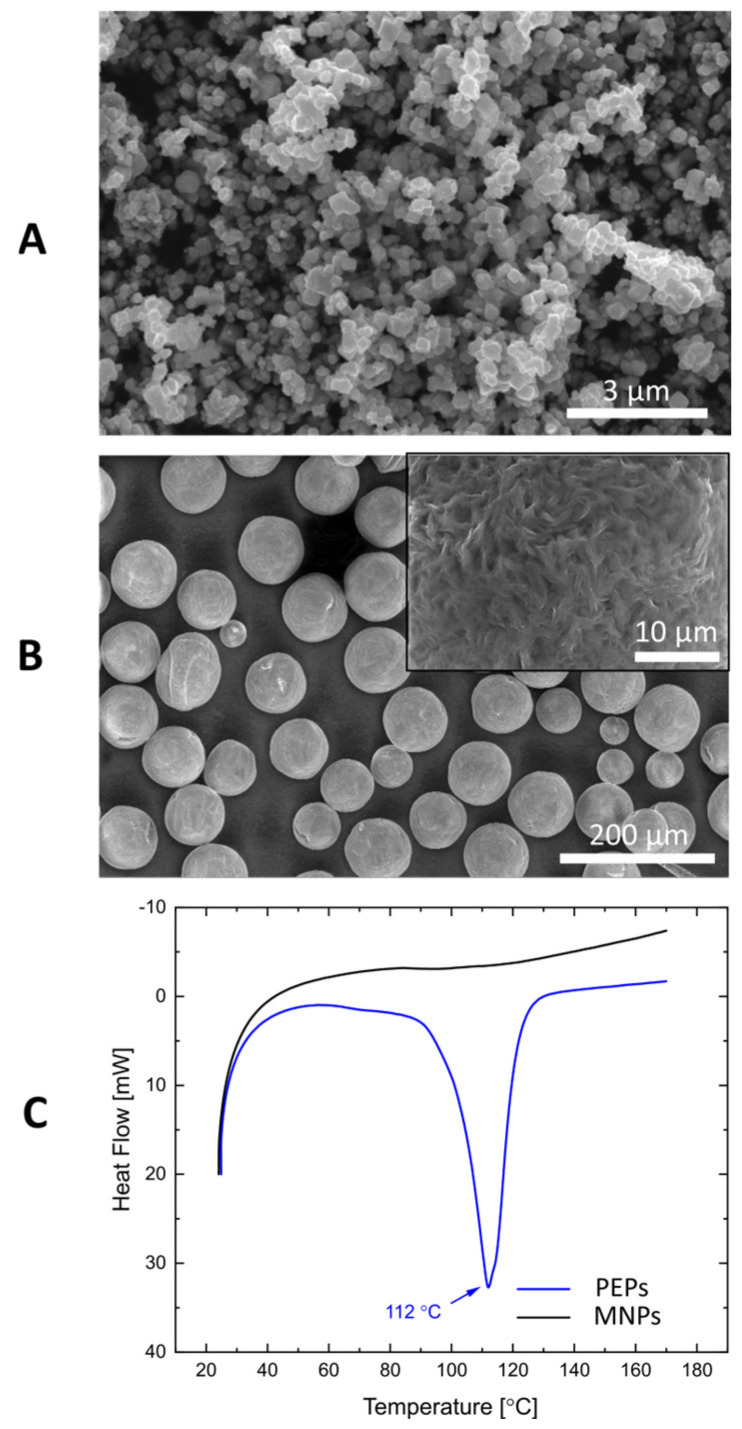
Characterization of particles used for the preparation of capsules. SEM images of (**A**) magnetic particles, MNPs, and (**B**) polyethylene particles, PEPs. The inset figure presents the magnified image of the single PE particle with a visible rough surface. (**C**) DSC results for both polyethylene and magnetic particles.

From these optical images, one can see that the capsule’s size reaches hundreds of micrometers, while the predicted size of the particle shell is also several tens of micrometers based on the polymer particle size shown in Figure 6B. Although these sizes are far too large for real applications in biomedicine, in the proposed method of formation, the final size of capsules could be altered by changing the size of stabilizing particles. Therefore, there is a possibility of scaling down the size of capsules to meet the criteria for usage in biomedical applications. For instance, the standard size of contrast agents in ultrasound imaging is around several micrometers [35]. Additionally, the smaller capsules created in the described way could also be used for this purpose, and further research on their contrasting properties could be done in the future. 

It is not only polymer particles that could be replaced with smaller ones. The change in the size of magnetic particles alters the heating properties and the resultant temperature increase since the type and size of particles, as well as the presence of the covering surfactant layer, influence much the heating properties during magnetic hyperthermia [36]. This is one of the important advantages of using magnetic heating over the application of the heat from outside, e.g., using a thermostated bath, and allows for the more controlled optimization of temperature inside the medium, for instance, in the case of usage of thermo-responsive materials with lower transition temperatures than polyethylene (Figure 6C). Moreover, the incorporation of magnetic nanoparticles in the structure of the capsule shell is desirable also because of the potential positioning of the capsules using external magnetic fields and the re-usage of magnetic heating of capsules when administered to the site of interest. 

## 4. Materials and Methods

### 4.1. Particles and Oils

This research used Rhodosil Oil 47V 50 silicone oil (VWR Chemicals, Radnor, PA, USA) as the dispersed phase and MA 220-1 castor oil (MERLIN, Logrono, Spain) as the continuous emulsion phase. Using these oils promotes system stabilization due to their similar density values. To stabilize the droplets, we used the polyethylene particles, CPMS-0.96 (Cospheric, Santa Barbara, CA, USA), as well as iron oxide magnetic particles (Nanografi Nano Technology, Ankara, Turkey). The size and shape of polyethylene particles and magnetic particles were characterized using an environmental scanning electron microscope (SEM) QUANTA 250 FEG, FEI (Hillsboro, OR, USA). Figure 6 shows the characterization of particles used to prepare capsules. The magnetic particles (Figure 6A) with a mean diameter <D> = 157.5 nm ± 3.7 nm presented superparamagnetic behavior (magnetization saturation: around 80 emu/g) [37]. The polymer particles were characterized with a rough surface and wide particle size distribution (10–90 μm), as shown in Figure 6B. The thermal properties of particles were revealed using differential scanning calorimetry (DSC). The DSC 8500 analyzer was provided by PerkinElmer Inc. (Waltham, MA, USA), and the temperature increase rate was 10 °C/min. Both tests were conducted in the Center for Advanced Research, AMU, Poznań, Poland. The magnetic nanoparticles did not exhibit the change during the experiment, while the transition temperature was determined from DSC results. It is 112 °C for polyethylene particles, as shown in Figure 6C. This temperature is close to the values of transition temperatures of polyethylene included in the literature [38,39,40].

### 4.2. Preparation of Pickering Emulsion-Based Capsules

First, the mixture of particles (magnetic and polyethylene) and oils was homogenized for 30 s using an ultrasonic device (acoustic intensity ≈ 17 W/cm^2^), resulting in the formation of a non-stable emulsion referred to as “pre-emulsion”, i.e., small silicone droplets partially covered by particles. Second, in the electric field, due to consecutive events of electrocoalescence, droplets formed during ultrasonication were gradually covered with particles up to complete surface coverage. In this way, we obtained Pickering “emulsions” [30]. The size of the droplets in Pickering emulsion prepared this way depends on the mass of the stabilizing particles and the mass of the dispersed oil phase used to create an emulsion [41]. We aimed to investigate systems containing droplets of different sizes. To achieve this, we prepared systems with three distinct mass ratios: 1:6, 1:12, and 1:24. The ratio was determined as the mass of polyethylene particles in relation to the mass of silicone oil used to create the system. While the masses of polyethylene (35 mg) and magnetite particles (48 mg) across all examined systems were constant, the mass of the silicone oil was systematically changed. 

Third, oil-in-oil Pickering emulsions stabilized by magnetic and polyethylene particles were exposed to the alternating magnetic field. Under the alternating magnetic field, magnetite particles acted as a good heat source in Pickering emulsions. Due to the Néel and Brown relaxation process and hysteresis losses [36,42], they became nano-heaters in an alternating magnetic field. Recently, magnetic heating was successfully used to induce a temperature rise in Pickering emulsions stabilized solely with iron oxide nanoparticles [43] and in Pickering droplets stabilized with magnetic and polymer particles to obtain sintered shells [2]. A compact induction heating system was used as a source of the alternating magnetic field (EASYHEAT, Ambrell Co., Rochester, NY, USA). The frequency of the field was 356 kHz, and the intensity was 16.2 kA/m.

**Figure 7:**
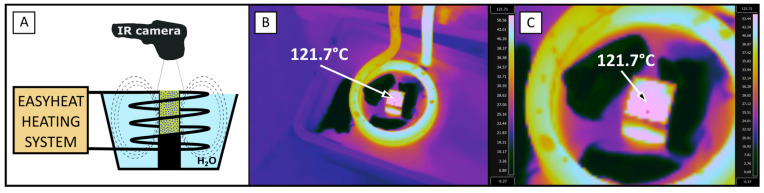
Temperature increase control during the process of magnetic heating under an alternating magnetic field. (**A**) Scheme drawing of the control setup; (**B**) thermal image of the heated system; and (**C**) close–up thermal image of the cuvette.

The temperature during magnetic heating was controlled in a non-invasive way using an infrared (IR) camera (FLIR e53, FLIR Co., Wilsonville, OR, USA). The scheme of the setup for thermography during the formation of capsules under an alternating magnetic field is shown in Figure 7. 

As one can see, the temperature obtained in the process of magnetic heating was much higher than the determined value of the transition temperature in Figure 6C (>112 °C), which suggests that the heated Pickering emulsions experienced structural changes. The IR camera detected the temperature of the sample solely on its surface. Due to the higher density of Pickering droplets compared to the castor oil, most of the magnetite particles were located in lower levels of the sample underneath the surface. As those particles were heat sources under the alternating magnetic field, the temperature inside the sample could have been higher than the one measured on the surface.

### 4.3. Ultrasound Measurement

**Figure 8:**
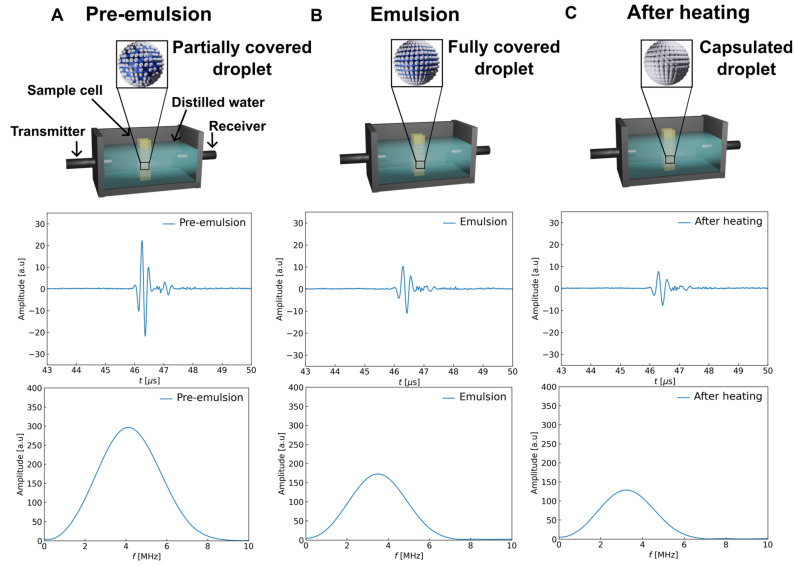
Schemes of the measuring system for control of Pickering emulsion-based capsule preparation by the ultrasonic method consist of an ultrasonic testing device with two piezoelectric broadband transducers, transmitter and receiver, immersed in a thermostatic sample cell at a temperature of 25 °C (**top row**). Ultrasound pulse (**middle row**) and the amplitude spectra (**bottom row**) for (**A**) pre-emulsion, (**B**) emulsion, and (**C**) the system after heating.

In this experiment, we used an ultrasonic spectroscopy technique based on transmission methods to characterize the Pickering emulsion-based capsule preparation. The ultrasonic measurements were carried out using two piezoelectric broadband transducers: a transmitter and a receiver (OLYMPUS, Waltham, MA, USA), driven by an ultrasound generator OPBOX 2.1 (OPTEL, Wrocław, Poland). Figure 8 presents the scheme for the ultrasound measurement system for the three stages of preparation (i.e., pre-emulsion, emulsion, and the colloidal system after magnetic heating). The ultrasound wave was transmitted through the water filling the container and sample cell (cuvette). Then, it was received by the receiver with a signal recorded at a sampling frequency of 100 MHz. The pulse signal waveform differed with different stages of preparation, as shown in Figure 8A–C (middle row). The amplitude loss occurs due to the more efficient attenuation of the wave after the tested systems change internal structure. The amplitude spectra were also calculated by applying the fast Fourier transformation (FFT) to each ultrasound pulse after filtering from unnecessary noise (bottom row). The attenuation of ultrasonic waves in the frequency domain was determined by analysis of the experimentally detected pulses using the following equation [44,45]:

(1) $$\alpha \left(f\right)={\alpha \left(f\right)}_{ref}+\frac{1}{d}\ln\frac{{|F}_{1}\left(f\right)|}{{|F}_{2}\left(f\right)|}.$$

Here, ${\alpha (f)}_{ref}=6.4\cdot f^{1.56}$ is the experimentally measured frequency-dependent attenuation coefficient (in Np/m) of the castor oil, where f is the frequency in MHz; *d* is the acoustic path inside the sample cell (0.01 m); $\left|F_{1}\right.\left.\left(f\right)\right|$ is the amplitude of the FFT for the pulse recorded in the carrier fluid; and $\left|F_{2}\right.\left.\left(f\right)\right|$ is the FFT amplitude of the pulse recorded in the pre-emulsion, emulsion, or after heating system, as shown in Figure 8A–C. 

The ultrasound phase velocity was determined using the following equation [46]:

(2) $$v=\frac{v_{0}2\pi df}{2\pi df-v_{0}(\theta_{1}\left(f\right)-\theta_{2}\left(f\right)+n2\pi )}.$$

Here, $v_{0}$ is the ultrasound velocity of the carrier fluid (castor oil), 1450 m/s; *f* is the frequency of the range from 1 MHz to 8 MHz; $\theta_{1}\left(f\right)$ is the FFT phase of the reference for the pulse recorded in the carrier liquid; $\theta_{1}\left(f\right)$ is the FFT phase; of the pulse recorded in the system; and *n* is the determined middle estimate from the calculated group velocity and comparison with phase velocity [47]. It is important to note that, in our experiment, we used a transmission method to determine phase velocity by referring the velocity of the tested system to the reference liquid, which was a continuous phase (castor oil). 

### 4.4. Optical Measurement

The microstructure of the emulsion droplets and capsules prepared for different ratios of particle mass to silicone oil mass was observed by a digital optical microscope (AM7115MZTL, Dino-Lite Europe, Almere, the Netherlands). The optical study was performed for freshly prepared systems using the same polystyrene cuvette that enabled direct measurement due to transparency. We used standard cuvettes for spectrophotometric measurements. 

## 5. Conclusions

In this work, we presented the potential application of ultrasound transmitted through the multi-phase system to characterize the structural changes during the formation of capsules from Pickering emulsion templates. The results showed that the propagation of ultrasound waves is sensitive to differences in oil droplet shell properties, which are reflected in changes in the ultrasound attenuation coefficient. By measuring the ultrasonic attenuation coefficient, we were able to characterize the whole process of droplet evolution, from pre-emulsion to the suspension of colloidal capsules. We showed experimentally that ultrasound was beneficial for studying colloidal capsule fabrication by magnetic heating using magnetite nanoparticles. The decrease in the ultrasonic wave amplitude after heating the system may be evidence of effective sintering of the particles on the droplet surface and, as a result, obtaining a suspension of colloidal capsules.

The attenuation coefficient analysis is a promising method of controlling the encapsulation process in Pickering emulsion-based systems, especially when the results are compared with optical images of investigated systems. Although the ultrasound velocity did not change significantly after the heating of the system, it could be used in the future to control temperature during the preparation process.

## Data Availability

The raw data supporting the conclusions of this article will be made available by the authors on request.
